# Assessment of a Text Message–Based Smoking Cessation Intervention for Adult Smokers in China: A Randomized Clinical Trial

**DOI:** 10.1001/jamanetworkopen.2023.0301

**Published:** 2023-03-01

**Authors:** Haoxiang Lin, Yihua Liu, Hao Zhang, Zhengjie Zhu, Xiaoyue Zhang, Chun Chang

**Affiliations:** 1 Institute for Global Health and Development, Peking University, Beijing, China; 2 Department of Social Medicine and Health Education, School of Public Health, Peking University Health Science Center, Beijing, China

## Abstract

**Importance:**

Successful smoking cessation strategies are an important part of reducing tobacco use. However, providing universal smoking cessation support can be a challenge for most countries because it requires sufficient resources. One way to expand access is to use mobile technologies to provide cessation support.

**Objective:**

To assess the efficacy of a behavior change theory–based smoking cessation intervention using personalized text messages.

**Design, Setting, and Participants:**

This study was a 2-arm double-blind randomized clinical trial conducted in 5 cities in China. Daily or weekly smokers 18 years or older were eligible for inclusion if they owned a mobile phone and used the WeChat social media app. A total of 722 participants were randomized to the intervention or control group between April 1 and July 27, 2021.

**Interventions:**

Intervention group participants received a personalized text message–based smoking cessation intervention that was based on the transtheoretical model and protection motivation theory and developed by this study’s investigators. Control group participants received a nonpersonalized text message–based smoking cessation intervention developed by the US National Cancer Institute. Both groups received 1 to 2 text messages per day for 3 months through the app.

**Main Outcomes and Measures:**

The primary outcome was the biochemically verified 6-month sustained abstinence rate, defined as the self-report of no smoking of any cigarettes after the designated quit date, which was validated biochemically by an expired air carbon monoxide level of less than 6 ppm at each follow-up point.

**Results:**

A total of 722 participants (mean [SD] age, 41.5 [12.7] years; 716 men [99.2%]; all of Chinese ethnicity) were randomly assigned to the intervention group (360 participants) or the control group (362 participants). Biochemically verified continuous abstinence at 6 months was 6.9% in the intervention group and 3.0% in the control group (odds ratio [OR], 2.66; 95% CI, 1.21-5.83). Among smokers with low nicotine dependence, the intervention group had significantly better abstinence rates for most of the indicators after adjusting for covariates (eg, biochemically verified 24-hour point prevalence of abstinence at 1 month: adjusted OR, 2.15; 95% CI, 1.05-4.38). Among smokers with moderate and high nicotine dependence, only the biochemically verified 24-hour point prevalence of abstinence at 6 months was statistically significant (adjusted OR, 4.17; 95% CI, 1.34-3.00). The pattern was similar for quitting intention, and the personalized text message–based intervention was more effective for smokers who had strong quitting intention than for those who had weak quitting intention.

**Conclusions and Relevance:**

In this study, the behavior change theory–based smoking cessation intervention using personalized text messages was more effective than an intervention using nonpersonalized text messages. The intervention was most effective among smokers with low nicotine dependence and strong quitting intention. This study’s findings also provide further evidence regarding the potential benefits of mobile health interventions for other behaviors.

**Trial Registration:**

Chinese Clinical Trial Registry Identifier: ChiCTR2100041942

## Key Points

**Question:**

What are the effects of a behavior change theory–based smoking cessation intervention using personalized text messages?

**Findings:**

In this randomized clinical trial of 722 current smokers in China, participants who received the behavior change theory–based smoking cessation intervention using personalized text messages had a 6-month quit rate that was twice that of participants who received an intervention using nonpersonalized text messages.

**Meaning:**

These findings provide new evidence supporting the utility of mobile health methods for smoking cessation.

## Teaser

This randomized clinical trial assesses the efficacy of a behavior change theory–based smoking cessation intervention using personalized text messages among adult smokers in China.

## Introduction

Emerging evidence supports the efficacy of mobile phone interventions for smoking cessation.^1,2,3^ Such interventions are easy to access and can actively reach smokers. The ubiquitous nature of mobile phones allows such interventions to provide effective and immediate help, increasing involvement and requiring low commitment. With the application of internet technologies, mobile phones can easily be used to deliver personalized interventions and tailored motivational messages.

The most common mobile smoking cessation programs have been based on messages sent through short messaging services or smartphone apps.^4^ Although both interventions have been confirmed to be effective to some extent,^4^ app-based messaging interventions are easier to personalize without incurring high costs and are not limited in terms of the number of text characters.

Many studies^4,5,6,7,8^ have found that mobile phones have the potential to provide smoking cessation support. An early randomized clinical trial (RCT)^5^ conducted by the London School of Hygiene and Tropical Medicine in 2011 showed that biochemically verified continuous abstinence at 6 months was significantly higher in an intervention group receiving text message support compared with a control group receiving no text message support (10.7% vs 4.9%). Another RCT^6^ evaluated the effect of messages sent through the WhatsApp platform and found that the use of WhatsApp messages in addition to usual care increased the abstinence rate approximately 2 times (OR, 2.31; 95% CI, 1.03-5.16) more than usual care alone over a 6-month period. Gram et al^7^ compared smoking cessation interventions delivered by mobile text messages and email and reported that smoking cessation interventions delivered by mobile text messages and email may be equally successful at the population level. There were also positive results from other social media platforms. For example, Cheung et al^8^ found that smokers who received counseling through Facebook had a lower relapse rate at 6 months than smokers in the control group who received a printed booklet. A Cochrane review^4^ found moderate-certainty evidence that automated text message–based smoking cessation interventions were associated with greater quit rates than minimal smoking cessation support and suggested that more RCTs were needed to assess these interventions.

China has a dual burden of smoking and limited health resources; however, mobile cessation interventions have neither been integrated into routine tobacco control programs nor attracted additional attention at the policy-maker level. An important reason for this gap in mobile interventions is the lack of a smoking cessation tool that has been proven effective for Chinese people via data from a large-sample study. We found only 2 previous studies^9,10^ related to mobile phone–based smoking cessation interventions targeting smokers in China. One study^9^ was conducted by the Chinese Center for Disease Control and Prevention, and it found that quit rates were high in both the high-frequency and low-frequency text messaging contact groups. However, that study^9^ only used self-reported point prevalence of abstinence and reported a high study withdrawal rate, which may have produced bias in the results. A more standard study (which included a biochemically verified quit rate, a higher follow-up rate, and a control group receiving no text message support)^10^ recruited 1295 adult smokers across 30 cities and provinces in China and found that biochemically verified continuous smoking abstinence at 24 weeks was 6.5% in the high-frequency text messaging group, 6.0% in the low-frequency text messaging group, and 1.9% in the control group; there was no difference in quit rates between the 2 intervention groups. However, the findings from that study^10^ were limited by the unblinded allocation of participants and the lack of a comparable control group.

The purpose of this study was to compare the efficacy of an investigator-developed behavior change theory–based smoking cessation intervention using personalized text messages (intervention group) with a US National Cancer Institute (NCI)–developed smoking cessation intervention using nonpersonalized text messages (control group).^9^ Both the intervention and control groups received messages through the WeChat app (the most popular Chinese social media app).

## Methods

### Study Design and Participants

This study was a 2-arm double-blind RCT conducted in 5 cities in China (Baotou, Beijing, Dezhou, Linzi, and Yakeshi). Participants were randomized to the intervention or control group between April 1 and July 27, 2021. Daily or weekly smokers 18 years or older were eligible for inclusion if they owned a mobile phone and used the app. Exclusion criteria included receipt of any smoking cessation treatment within 30 days and a diagnosis of any mental illness (current or past). This trial was reviewed and approved by the ethics committee of the Peking University Health Science Center. The trial protocol is provided in Supplement 1; no changes from the original proposal in terms of methods or outcome measures occurred after the trial began. All participants signed informed consent forms before randomization and knew they could withdraw from the study at any time. All patient information was accessible only to personnel participating in the study. For the statistical analysis, all patient information was encoded to guarantee that personal information would be unidentifiable during the processing of the data and the reporting of the results. We did not provide financial compensation to participants, but gifts (a towel, umbrella, or cup) were given to participants if they completed 1 follow-up visit. This study followed the Consolidated Standards of Reporting Trials (CONSORT) reporting guideline for RCTs.

### Procedures

#### Development of the Intervention Framework and Text Message Bank

The theoretical framework of the intervention was based on the transtheoretical model (TTM) and the protection motivation theory (PMT). Both models have been independently applied to health behavior change interventions.^11,12^ For the TTM, given the systematic relationship between the stages and processes of change, several strategies were used to strengthen behavior change and/or to achieve the next stage.

As determined using the app, smokers’ quitting intention to try to quit in the next 6 months was assessed on a 5-point scale, with 1 indicating not at all likely and 5 indicating very likely; a score of 1 to 3 points was considered a weak quitting intention, and 4 to 5 points was considered a strong quitting intention

On the prequit date, messages related to consciousness raising, dramatic relief, and environmental reevaluation were provided to smokers with weak quitting intention. Messages related to stimulus control, self-liberation, and reinforcement management were provided to smokers with strong quitting intention. On the early quit and late quit dates, messages related to consciousness raising, dramatic relief, environmental reevaluation, and self-reevaluation were sent to smokers who had relapsed. Smokers who remained abstinent only received information based on the PMT.

The text message bank was created using messages developed by the Peking University School of Public Health with the input of smokers and smoking cessation professionals. The messages had a 3-layer framework. The first layer was divided based on time and consisted of a prequit message, quit day message, withdrawal symptom management message, early quit message, and late period message. The second layer was divided based on the TTM, and the third layer was divided based on the PMT. The core motivational messages consisted of 14 subgroups with a total of 200 text messages; there were also approximately 200 contact messages. A more detailed description of the intervention framework based on the TTM and PMT and additional information about the text message bank are available in the eTable and eMethods in Supplement 2.

#### Recruitment

We marketed the trial to smokers through print advertising (leaflets), digital advertising (the app), and staff from Shougang Technical College (teachers and leaders). Potential participants contacted the local Disease Control and Prevention Center to register their interest. The research assistants explained the study to each potential participant and preliminarily assessed his or her eligibility. All eligible smokers were told they needed to come to a location (typically the meeting room of the Disease Control and Prevention Center or a hotel) on a fixed date to finalize the recruitment process. Participant eligibility was confirmed, and all participants signed an informed consent form during the first face-to-face contact.

#### Randomization and Blinding

After recruitment, participants were required to register through the app. A total of 780 current smokers were screened (Figure). We excluded 43 potential participants for 3 reasons. First, they were monthly smokers rather than daily or weekly smokers. Second, their cellular phones could not satisfy the requirement. Third, they could not complete the long-term follow-up. A total of 737 participants were randomized; of those, 15 withdrew from the study (refused randomization). Smoking cessation results were available for 722 participants who completed randomization; of those, 360 were randomized to the intervention group, and 362 were randomized to the control group. This sample size allowed a power of 80% to detect a modest difference between each group, with a significance level of α = .05. Overall, 700 participants completed follow-up at 6 months (97.0% retention rate); all of the 22 participants who did not complete follow-up declined interviews and were counted as smokers. Therefore, the primary outcome had no unavailability for follow-up.

**Figure.**
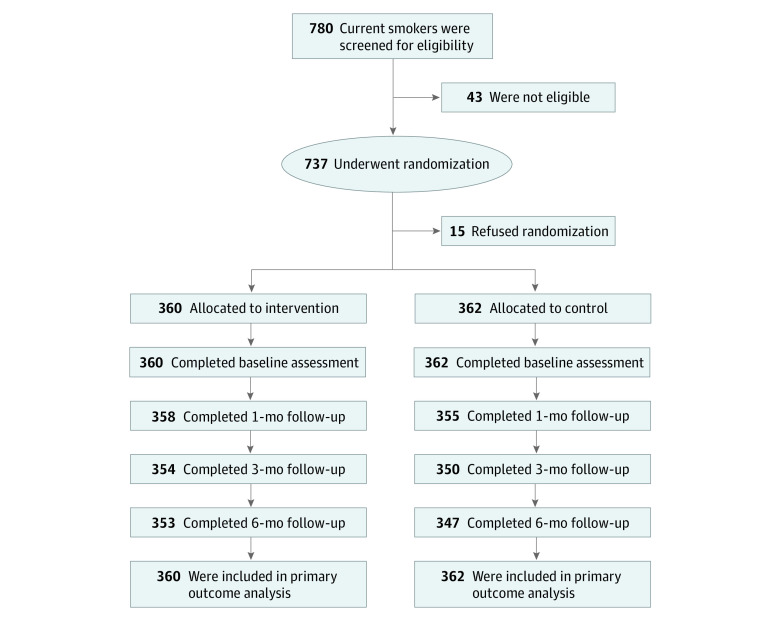
Study Flowchart

A randomized block design was used, and the participant score on the Fagerström Test for Nicotine Dependence (range, 0-10, with higher scores indicating greater dependence)^13^ was treated as a stratified factor. The app automatically generated 2 blocks based on test score, with block 1 comprising participants with low or moderate nicotine dependence (score of 0-6 points) and block 2 comprising participants with high nicotine dependence (score of ≥7 points).^13^ Eligible participants were assigned to the intervention group or the control group within each block by simple randomization. Independent information technology personnel created a randomization sequence but were not involved in the research. The app was also used to balance demographic characteristics. Randomization was fully computerized and automated with equal allocation. The researchers and participants were all blinded to randomization. After randomization, participants were required to complete the baseline questionnaire.

#### Intervention

All of the participants were informed that the eighth day after randomization would be their quit day. Participants who were randomized to the intervention group received the program of interventions (1-2 personalized text messages per day for 3 months).

Control group participants received a smoking cessation intervention that used nonpersonalized text messages and was developed by the NCI. The NCI intervention was based on well-established cognitive-behavioral cessation approaches and contained 91 messages. Participants received approximately 1 message per day for 3 months. The text messages provided encouragement, practical advice to help maintain cessation, and information on the health effects of smoking. The details of the control intervention can be found elsewhere.^9^

#### Assessment

All participants in the 2 groups were asked to attend face-to-face follow-up visits with research staff (H.L., Y.L., H.Z., Z.Z., and X.Z.) at 1 month, 3 months, and 6 months after randomization. The primary outcome was the biochemically verified 6-month sustained abstinence rate, defined as the self-report of no smoking of any cigarettes after the designated quit date, which was validated biochemically by an expired air carbon monoxide level of less than 6 ppm at each follow-up point.^14^ Research has confirmed that expired air carbon monoxide tests with a cutoff of 6 ppm have 97% sensitivity and 70% specificity in normal environments.^15^ Therefore, expired air carbon monoxide test levels have become a recommended biochemical verification method for detecting recent smoking.

Secondary outcomes were biochemically verified sustained abstinence rates at 1 month, 3 months, 1 to 3 months, and 3 to 6 months after randomization; biochemically verified 24-hour point prevalence of abstinence at each follow-up point; self-reported sustained abstinence rate; 24-hour point prevalence of abstinence at each follow-up point; change in nicotine dependence measured by the Fagerström Test for Nicotine Dependence^13^; change in nicotine withdrawal symptoms measured by the Minnesota Nicotine Withdrawal Scale (which evaluates the symptom severity of cigarette cravings, irritability, anxiety, difficulty concentrating, restlessness, headache, drowsiness, and gastrointestinal tract disturbances)^16^; and change in the readings of expired air carbon monoxide.

All face-to-face follow-up visits, which included administration of all biochemical verification tests and questionnaires, were conducted by staff members of the local Disease Control and Prevention Center and Peking University. Most of the meetings took place in rooms at the Disease Control and Prevention Center, but some staff members met participants in their homes or any place convenient for the participants.

### Statistical Analysis

Descriptive statistics were calculated to compare the baseline status between the 2 groups. We conducted intention-to-treat and Russell Standard analyses for each group. All randomized participants were included in the denominator for calculating abstinence rates, with the exception of participants who were unavailable for follow-up for unavoidable reasons (ie, the participant died or moved to an untraceable address). Those who declined to be involved in subsequent data collection were counted as smokers.^14^ This type of analysis is considered the most conservative and is the standard for smoking cessation studies.^10^

The primary and secondary abstinence outcomes were analyzed using logistic regression analysis of smoking status at each time point. The dependent variable was smoking status (with 0 indicating still smoking and 1 indicating abstinent). The independent variable was each intervention condition vs the control condition. Odds ratios (ORs) were used to measure the outcomes for the intervention group compared with the control group, and 95% CIs and *P* values were calculated to infer efficacy. In sensitivity analyses, each model was further adjusted for the following baseline covariates: living area, age, educational level, smoking frequency, and nicotine dependence. All baseline covariates used in these analyses were categorical variables.

We compared the change in nicotine dependence, nicotine withdrawal symptoms, and expired air carbon monoxide readings during the intervention and follow-up periods between the 2 groups using descriptive statistics and 2-sample *t* tests. The analysis of withdrawal symptoms was restricted to participants who were abstinent since the previous visit. The analysis of change in nicotine dependence was restricted to those who did not quit smoking.

A threshold of 2-sided *P* < .05 was used to determine statistical significance. All analyses were performed using IBM SPSS Statistics, version 11.0 (IBM Corporation).

## Results

### Participants

Among 722 participants, the mean (SD) age was 41.5 (12.7) years. Most of the study sample was male (716 of 722 individuals [99.2%]) and had an educational level of college or higher (440 of 721 individuals [61.0%]) (Table 1). All participants were Chinese; 677 (93.8%) were of Han ethnicity, and 45 (6.2%) were of other ethnicities. A total of 453 of 704 participants (64.3%) lived in urban areas. The sample was largely composed of daily smokers (592 of 720 individuals [82.2%]), with only 128 of 720 individuals (17.8%) being weekly smokers. The intervention and control groups were well balanced with respect to baseline demographic and smoking-related characteristics.

**Table 1.** Baseline Participant Characteristics

Characteristic	Participants, No./total No. (%)			χ^2^ Statistic	*P* value
	Total (N = 722)	Control group (n = 362)	Intervention group (n = 360)		
Sex					
Male	716/722 (99.2)	358/362 (98.9)	358/360 (99.4)	0.66	.42
Female	6/722 (0.8)	4/362 (1.1)	2/360 (0.6)		
Age, y					
18-44	396/722 (54.8)	212/362 (58.6)	184/360 (51.1)	4.05	.13
45-64	311/722 (43.1)	143/362 (39.5)	168/360 (46.7)		
>64	15/722 (2.1)	7/362 (1.9)	8/360 (2.2)		
Educational level					
Middle school or lower	112/721 (15.5)	54/362 (14.9)	58/359 (16.2)	5.31	.07
High school	169/721 (23.4)	73/362 (20.2)	96/359 (26.7)		
College or higher	440/721 (61.0)	235/362 (64.9)	205/359 (57.1)		
Ethnicity					
Han	677/722 (93.8)	341/362 (94.2)	336/360 (93.3)	0.23	.63
Other	45/722 (6.2)	21/362 (5.8)	24/360 (6.7)		
Living area					
Urban	453/704 (64.3)	230/349 (65.9)	223/355 (62.8)	0.73	.39
Rural	251/704 (35.7)	119/349 (34.1)	132/355 (37.2)		
Smoking status					
Daily	592/720 (82.2)	293/360 (81.4)	299/360 (83.1)	0.34	.56
Weekly	128/720 (17.8)	67/360 (18.6)	61/360 (16.9)		
No. of cigarettes smoked per day for daily smokers					
<5	36/592 (6.1)	25/293 (8.5)	11/299 (3.7)	8.37	.08
5-9	95/592 (16.0)	46/293 (15.7)	49/299 (16.4)		
10-14	163/592 (27.5)	76/293 (25.9)	87/299 (29.1)		
15-24	250/592 (42.2)	127/293 (43.3)	123/299 (41.1)		
>24	48/592 (8.1)	19/293 (6.5)	29/299 (9.7)		
No. of cigarettes smoked per week for weekly smokers					
<5	39/128 (30.5)	26/67 (38.8)	13/61 (21.3)	5.71	.22
5-9	21/128 (16.4)	11/67 (16.4)	10/61 (16.4)		
10-14	15/128 (11.7)	6/67 (9.0)	9/61 (14.8)		
15-24	25/128 (19.5)	10/67 (14.9)	15/61 (24.6)		
>24	28/128 (21.9)	14/67 (20.9)	14/61 (23.0)		
Monthly income, ¥/$					
<4000/<573	346/722 (47.9)	177/362 (48.9)	169/360 (46.9)	0.36	.84
4000-5999/573-859	231/722 (32.0)	115/362 (31.8)	116/360 (32.2)		
≥6000/≥860	145/722 (20.1)	70/362 (19.3)	75/360 (20.8)		
Nicotine dependence					
Low	456/717 (63.6)	228/360 (63.3)	228/357 (63.9)	0.14	.93
Moderate	202/717 (28.2)	101/360 (28.1)	101/357(28.3)		
High	59/717 (8.2)	31/360 (8.6)	28/357 (7.8)		
Quitting intention					
Strong	537/722 (74.4)	265/362 (73.2)	272/360 (75.6)	0.52	.47
Weak	185/722 (25.6)	97/362 (26.8)	88/360 (24.4)		

### Outcomes

Regarding the primary outcome, biochemical continuous abstinence at 6 months was verified for 25 of 360 participants (6.9%) in the intervention group and 11 of 362 participants (3.0%) in the control group (OR, 2.38; 95% CI, 1.15-4.92; adjusted OR [AOR], 2.66; 95% CI, 1.21-5.83) (Table 2). Regarding secondary outcomes, biochemically verified continuous abstinence at 1 month (AOR, 2.13; 95% CI, 1.16-3.92), 3 months (AOR, 2.80; 95% CI, 1.35-5.82), 1 to 3 months (AOR, 2.34; 95% CI, 1.29-4.24), and 3 to 6 months (AOR, 2.24; 95% CI, 1.32-3.81) after randomization and biochemically verified 24-hour point prevalence of abstinence at 1 month (AOR, 2.13; 95% CI, 1.16-3.92) and 6 months (AOR, 1.89; 95% CI, 1.19-3.02) after randomization were significantly better in the intervention group than in the control group, except for the 24-hour point prevalence of abstinence at 3 months (AOR, 1.30; 0.81-2.09). The biochemically verified 24-hour point prevalence of abstinence increased over time in both groups; however, the increase was slower in the control group vs the intervention group after the intervention ended (ie, 3 months after randomization). The results were similar for self-reported indicators, and the intervention group had significantly better self-reported abstinence rates, except for the 24-hour point prevalence of abstinence at 3 months.

**Table 2.** Abstinence Rates at Different Time Points

Outcome	Participants, No. (%)		OR (95% CI)	*P* value	Sensitivity analysis, AOR (95% CI)^a^	*P* value	NNT
	Intervention group (n = 360)	Control group (n = 362)					
**Primary outcome**							
Biochemically verified continuous abstinence at 6 mo	25 (6.9)	11 (3.0)	2.38 (1.15-4.92)	.02	2.66 (1.21-5.83)	.02	26
**Secondary outcomes**							
Biochemically verified continuous abstinence							
1 mo	37 (10.3)	21 (5.8)	1.86 (1.07-3.25)	.03	2.13 (1.16-3.92)	.02	22
3 mo	30 (8.3)	13 (3.6)	2.44 (1.25-4.76)	<.001	2.80 (1.35-5.82)	<.001	21
1-3 mo	42 (11.7)	21 (5.8)	2.15 (1.24-3.70)	<.001	2.34 (1.29-4.24)	<.001	17
3-6 mo	53 (14.7)	28 (7.7)	2.06 (1.27-3.34)	<.001	2.24 (1.32-3.81)	<.001	14
Biochemically verified 24-h point prevalence of abstinence							
1 mo	37 (10.3)	21 (5.8)	1.86 (1.07-3.25)	.03	2.13 (1.16-3.92)	.02	22
3 mo	56 (15.6)	45 (12.4)	1.30 (0.85-1.98)	.23	1.30 (0.81-2.09)	.27	31
6 mo	73 (20.3)	48 (13.3)	1.67 (1.12-2.48)	.01	1.89 (1.19-3.02)	<.001	14
Self-reported continuous abstinence							
1 mo	43 (11.9)	26 (7.2)	1.75 (1.05-2.92)	.03	2.17 (1.23-3.82)	<.001	21
3 mo	36 (10.0)	16 (4.4)	2.40 (1.31-4.41)	<.001	3.03 (1.54-5.97)	<.001	18
6 mo	33 (9.2)	14 (3.9)	2.51 (1.32-4.77)	<.001	3.12 (1.53-6.34)	<.001	19
1-3 mo	51 (14.2)	30 (8.3)	1.83 (1.13-2.94)	.01	2.07 (1.22-3.52)	<.001	17
3-6 mo	63 (17.5)	36 (9.9)	1.92 (1.23-2.98)	<.001	2.11 (1.30-3.43)	<.001	13
Self-reported 24-h point prevalence of abstinence							
1 mo	43 (11.9)	26 (7.2)	1.75 (1.05-2.92)	.03	2.17 (1.23-3.82)	<.001	21
3 mo	78 (21.7)	60 (16.6)	1.39 (0.96-2.02)	.08	1.50 (0.99-2.26)	.06	20
6 mo	96 (26.7)	66 (18.2)	1.63 (1.14-2.33)	<.001	1.91 (1.27-2.89)	<.001	12

Abbreviations: AOR, adjusted odds ratio; NNT, number needed to treat; OR, odds ratio.

^a^ The analysis was adjusted for living area, age, educational level, smoking frequency, and nicotine dependence at baseline.

The effects of the intervention on withdrawal symptoms and changes in expired air carbon monoxide readings and nicotine dependence are shown in the eFigure in Supplement 2. We also stratified the sample by nicotine dependence level and quitting intention at baseline. We found that our intervention was more effective for smokers with low nicotine dependence than for smokers with moderate and high nicotine dependence (Table 3). For smokers with low nicotine dependence, we found that the intervention group had significantly better abstinence rates for most of the indicators after adjusting for covariates (eg, biochemically verified 24-hour point prevalence of abstinence at 1 month: AOR, 2.15; 95% CI, 1.05-4.38). For smokers with moderate and high nicotine dependence, the intervention group had a higher proportion of abstinence rates for all indicators; however, only the biochemically verified 24-hour point prevalence of abstinence at 6 months was statistically significant (AOR, 4.17; 95% CI, 1.34-3.00). The pattern was similar for quitting intention, and we found that our intervention was more effective for smokers who had strong quitting intention than for those who had weak quitting intention (Table 4).

**Table 3.** Abstinence Rates at Different Time Points Stratified by Nicotine Dependence Level

Outcome	Participants, No./total No. (%)		OR (95% CI)	*P* value	Sensitivity analysis, AOR (95% CI)^a^	*P* value
	Intervention group	Control group				
**Low nicotine dependence: primary outcome**						
Biochemically verified continuous abstinence at 6 mo	17/228 (7.5)	9/228 (3.9)	1.96 (0.86-4.50)	.11	2.10 (0.87-5.08)	.10
**Low nicotine dependence: secondary outcomes**						
Biochemically verified continuous abstinence						
1 mo	27/228 (11.8)	16/228 (7.0)	1.78 (0.93-3.40)	.08	2.15 (1.05-4.38)	.04
3 mo	21/228 (9.2)	10/228 (4.4)	2.21 (1.01-4.81)	.05	2.58 (1.11-5.80)	.03
Biochemically verified 24-h point prevalence of abstinence						
1 mo	27/228 (11.8)	16/228 (7.0)	1.78 (0.92-3.04)	.08	2.15 (1.05-4.38)	.04
3 mo	41/228 (18.0)	37/228 (16.2)	1.13 (0.70-1.84)	.62	1.20 (0.70-2.07)	.50
6 mo	53/228 (23.2)	41/228 (18.0)	1.38 (0.88-2.18)	.17	1.54 (0.91-2.62)	.11
Self-reported continuous abstinence						
1 mo	33/228 (14.5)	18/228 (7.9)	1.97 (1.08-3.62)	.03	2.35 (1.22-4.54)	.01
3 mo	26/228 (11.4)	11/228 (4.8)	2.54 (1.22-5.27)	.01	3.15 (1.42-7.00)	<.001
6 mo	25/228 (11.0)	9/228 (3.9)	3.00 (1.37-6.57)	<.001	3.56 (1.54-8.23)	<.001
Self-reported 24-h point prevalence of abstinence						
1 mo	33/228 (14.5)	18/228 (7.9)	1.97 (1.08-3.62)	.03	2.35 (1.22-4.54)	.01
3 mo	57/228 (25.0)	47/228 (20.6)	1.28 (0.83-1.99)	.27	1.44 (0.88-2.34)	.14
6 mo	73/228 (32.0)	52/228 (22.8)	1.60 (1.05-2.42)	.03	1.90 (1.17-3.07)	<.001
**Moderate and high nicotine dependence: primary outcome**						
Biochemically verified continuous abstinence at 6 mo	7/129 (5.4)	2/132 (1.5)	3.73 (0.76-18.30)	.11	6.00 (0.86-41.78)	.07
**Moderate and high nicotine dependence: secondary outcomes**						
Biochemically verified continuous abstinence						
1 mo	9/129 (7.0)	5/132 (3.8)	1.91 (0.62-5.85)	.26	2.21 (0.65-7.52)	.20
3 mo	8/129 (6.2)	3/132 (2.3)	2.84 (0.74-10.97)	.13	3.41 (0.72-16.08)	.12
Biochemically verified 24-h point prevalence of abstinence						
1 mo	9/129 (7.0)	5/132 (3.8)	1.91 (0.62-5.85)	.26	2.21 (0.65-7.52)	.20
3 mo	13/129 (10.1)	8/132 (6.1)	1.74 (0.70-4.34)	.24	1.91 (0.67-5.45)	.23
6 mo	19/129 (14.7)	7/132 (5.3)	3.08 (1.25-7.61)	.02	4.18 (1.34-13.03)	.01
Self-reported continuous abstinence						
1 mo	9/129 (7.0)	8/132 (6.1)	1.16 (0.43-3.11)	.76	1.81 (0.58-5.67)	.31
3 mo	9/129 (7.0)	5/132 (3.8)	1.91 (0.62-5.85)	.26	2.83 (0.75-10.68)	.12
6 mo	7/129 (5.4)	5/132 (3.8)	1.46 (0.45-4.72)	.53	2.13 (0.53-8.58)	.29
Self-reported 24-hour point prevalence of abstinence						
1 mo	9/129 (7.0)	8/132 (6.1)	1.16 (0.43-3.11)	.76	1.81 (0.58-5.67)	.31
3 mo	19/129 (14.7)	13/132 (9.8)	1.58 (0.75-3.35)	.23	1.85 (0.80-4.28)	.15
6 mo	22/129 (17.1)	14/132 (10.6)	1.73 (0.84-3.56)	.13	2.06 (0.91-4.68)	.09

Abbreviations: AOR, adjusted odds ratio; OR, odds ratio.

^a^ The analysis was adjusted for living area, age, educational level, smoking frequency, and nicotine dependence at baseline.

**Table 4.** Abstinence Rates at Different Time Points Stratified by Quitting Intention at Baseline

Outcome	Participants, No./total No. (%)		OR (95% CI)	*P* value	Sensitivity analysis, AOR (95% CI)^a^	*P* value
	Intervention group	Control group				
**Strong quitting intention: primary outcome**						
Biochemically verified continuous abstinence at 6 mo	24/272 (8.8)	11/265 (4.2)	2.24 (1.07-4.66)	.03	2.51 (1.13-5.54)	.02
**Strong quitting intention: secondary outcomes**						
Biochemically verified continuous abstinence						
1 mo	35/272 (12.9)	20/265 (7.5)	1.81 (1.02-3.22)	.04	2.06 (1.09-3.88)	.03
3 mo	29/272 (10.7)	12/265 (4.5)	2.52 (1.26-5.04)	<.001	2.97 (1.38-6.36)	<.001
Biochemically verified 24-h point prevalence of abstinence						
1 mo	35/272 (12.9)	20/265 (7.5)	1.81 (1.02-3.22)	.04	2.06 (1.09-3.88)	.03
3 mo	53/272 (19.5)	40/265 (15.1)	1.36 (0.87-2.14)	.18	1.35 (0.81-2.24)	.24
6 mo	67/272 (24.6)	42/265 (15.8)	1.74 (1.13-2.67)	.01	1.94 (1.18-3.21)	<.001
Self-reported continuous abstinence						
1 mo	41/272 (15.1)	24/265 (9.1)	1.78 (1.04-3.04)	.03	2.21 (1.22-4.02)	<.001
3 mo	35/272 (12.9)	15/265 (5.7)	2.46 (1.31-4.62)	<.001	3.18 (1.57-6.46)	<.001
6 mo	32/272 (11.8)	13/265 (4.9)	2.59 (1.33-5.04)	<.001	3.28 (1.56-6.88)	<.001
Self-reported 24-h point prevalence of abstinence						
1 mo	41/272 (15.1)	24/265 (9.1)	1.78 (1.04-3.04)	.03	2.21 (1.22-4.02)	<.001
3 mo	71/272 (26.1)	53/265 (20.0)	1.41 (0.94-2.12)	.09	1.49 (0.95-2.35)	.08
6 mo	86/272 (31.6)	58/265 (21.9)	1.65 (1.12-2.43)	.01	1.91 (1.22-2.99)	<.001
**Weak quitting intention: primary outcome**						
Biochemically verified continuous abstinence at 6 mo	1/88 (1.1)	0	NA	NA	NA	NA
**Weak quitting intention: secondary outcomes**						
Biochemically verified continuous abstinence						
1 mo	2/88 (2.3)	1/97 (1.0)	2.23 (0.20-25.06)	.52	1.86 (0.13-27.09)	.65
3 mo	1/88 (1.1)	1/97 (1.0)	1.10 (0.07-17.91)	.95	NA	NA
Biochemically verified 24-h point prevalence of abstinence						
1 mo	2/88 (2.3)	1/97 (1.0)	2.23 (0.20-25.06)	.52	1.86 (0.13-27.09)	.65
3 mo	5/88 (5.7)	3/97 (3.1)	0.65 (0.15-2.80)	.56	0.64 (0.12-3.42)	.64
6 mo	6/88 (6.8)	6/97 (6.2)	1.10 (0.34-3.58)	.86	1.33 (0.31-5.74)	.70
Self-reported continuous abstinence						
1 mo	2/88 (2.3)	2/97 (2.1)	1.11 (0.15-8.01)	.92	1.09 (0.13-8.85)	.94
3 mo	1/88 (1.1)	1/97 (1.0)	1.10 (0.07-17.91)	.95	NA	NA
6 mo	1/88 (1.1)	1/97 (1.0)	1.10 (0.07-17.91)	.95	NA	NA
Self-reported 24-h point prevalence of abstinence						
1 mo	2/88 (2.3)	2/97 (2.1)	1.11 (0.15-8.01)	.92	1.09 (0.13-8.85)	.94
3 mo	7/88 (8.0)	7/97 (7.2)	1.11 (0.37-3.30)	.85	1.21 (0.33-4.40)	.77
6 mo	10/88 (11.4)	8/97 (8.2)	1.43 (0.54-3.79)	.48	1.62 (0.51-5.09)	.41

Abbreviations: AOR, adjusted odds ratio; NA, not applicable; OR, odds ratio.

^a^ The analysis was adjusted for living area, age, educational level, smoking frequency, and nicotine dependence at baseline.

During the trial, other smoking cessation support and/or services were used by 77 of 360 participants (21.4%) in the intervention group vs 91 of 362 participants (25.1%) in the control group. This difference was not statistically significant.

## Discussion

This RCT showed that the behavior change theory–based smoking cessation intervention using personalized text messages was more effective than an intervention using nonpersonalized text messages in both the short and long term and should be considered as an intervention to aid in quitting smoking nationwide. With these findings, the current study joined the recent debate on the effect of mobile interventions on smoking cessation and provided evidence using a robust study design. The study also found new and potentially important information. The primary outcome, biochemically verified 6-month sustained abstinence rate, was relatively low (6.9% in the intervention group) compared with that of Western countries. For example, a British mobile phone text messaging study^5^ found that biochemically verified continuous abstinence at 6 months was 10.7%. Some other studies^7,17^ reported a higher quit rate. A possible explanation for the discrepancy with our findings is that the social environment in China may have a negative influence on quitting smoking. Smoking is not only a habit but part of the traditional culture and social interaction in China, similar to tea culture, which is deeply rooted in people’s daily lives.^18^ Therefore, it is unsurprising that a smoking cessation intervention had a lower abstinence rate in China.

Notably, the biochemically verified 24-hour point prevalence of abstinence increased in both groups; however, the increase became slower in the control group than the intervention group after the intervention ended at 3 months. This slower increase may be due to the fact that the personalized intervention improved efficacy and affected the duration of the intervention. Research has found that personalized messages elicit neural activity in self-processing regions, and this self-related activity estimates the success of smoking cessation.^19^ This finding is encouraging for study interventions such as ours because it suggests that the effect of a personalized smoking cessation intervention can continue after a study ends and may motivate self-intervention that is much more meaningful for practice.

We did not provide financial compensation to the participants; however, we provided gifts (a towel, umbrella, or cup) if the participants completed 1 follow-up visit. Our study had a high retention rate, probably due to 3 reasons. First, at the time of the baseline survey, we recorded participants’ full information, including home address, telephone number, family member’s telephone number, and company or organization of employment. If participants could not come to our designated location to complete a follow-up visit, we sent a research staff member to any location of their convenience (normally their home or place of employment). Second, if we could not contact participants by cell phone, we contacted their family member to coordinate a time to complete the follow-up visit. Third, if we tried everything but still could not locate a participant, the local Disease Control and Prevention Center helped us to connect with their community service center to find the individual and complete the follow-up visit. All procedures were explained to participants at baseline.

Our findings have policy implications. This study was, to our knowledge, the first double-blind RCT of a mobile smoking cessation intervention in mainland China. On the basis of the study results and because population-level smoking cessation services are limited in China, a mobile cessation intervention should be considered as an addition to existing smoking cessation services. Furthermore, given the low cost of such an intervention, the delivery of interventions at the national or international level would be easy and especially beneficial for countries such as China that have a large number of smokers but no cost-free smoking cessation medications and support.

Our study also has theoretical implications; mobile interventions are often limited by insufficient reporting of content, which leaves other researchers unclear regarding which specific factors made an intervention effective. In the context of mobile smoking cessation interventions, inadequate reporting is of particular concern. The current study provides a clear framework for the intervention through the systematic and transparent application of the PMT and TTM. This approach may allow other researchers to benefit from our experience when scaling up their smoking cessation interventions, and the methods used may be equally applicable to the development of interventions targeting other health behaviors.

Further trials are needed to determine whether our results generalize to female and younger populations. In addition, studies that compare a mobile smoking cessation intervention with an intervention consisting of both mobile cessation and other counseling services are needed. These studies will be important for public health policy decisions regarding whether health care organizations should provide a mobile cessation intervention as a treatment option for smokers or integrate mobile cessation into a current service.

### Strengths and Limitations

This study has several strengths. Use of the app for randomization ensured that the researchers and participants had neither foreknowledge of treatment allocation nor the risk of identification during the intervention. Baseline demographic and smoking-related characteristics were well balanced between groups. The rigorous measurements of abstinence with biochemical verification suggested by the Russell Standard were applied in this study. Participants who declined to be involved in subsequent data collection were counted as smokers; therefore, the outcome findings in this study reflected somewhat conservative estimates. The transparency and clear framework of the intervention may allow future researchers to take advantage of our experiences.

The study also has limitations. First, although efforts were made to ensure that both researchers and participants remained blinded to the randomization, a risk of breaking the blinding was present. For example, if a participant reported a problem with his or her program, the researchers needed to contact the information technology staff to investigate the problem, and they had the opportunity to identify the participant’s allocated group. Second, expired air carbon monoxide testing is the common method of detecting recent smoking and is recommended as a standard for the assessment of smoking cessation in trials. However, these biochemical tests are not perfect; expired air carbon monoxide can be detected only for approximately 24 hours after tobacco use. Bias can occur if a smoker stops smoking before providing a sample for testing, then resumes smoking after testing but reports that they have stopped smoking. Third, most of the participants were male and older than 18 years; therefore, caution should be used when generalizing our findings to the population level, as heterogeneity may exist. Fourth, although both groups had the same intervention period, the intervention group occasionally received 2 messages per day (vs 1 message per day in the control group) based on the framework of the intervention design. Therefore, the positive outcome of this study may be due to the different frequencies of contact. Fifth, there were only 6 female smokers in the cohort. Therefore, the study’s results might not be fully representative of female smokers.

## Conclusions

This RCT found that a behavior change theory–based intervention using personalized text messages increased biochemically verified smoking cessation at 6 months compared with an intervention using nonpersonalized text messages. This study provided new evidence supporting the utility of mobile health methods for smoking cessation. Overall, this research not only contributes to the development of a feasible smoking cessation intervention but also provides further evidence regarding the potential benefits of mobile health interventions for other behaviors, adding to previous research on this topic.

## Notes

Supplement 1.
Trial Protocol

Supplement 2. **eTable.** Theoretical Framework of the Intervention **eMethods.** Development of the Intervention Framework and the Text Bank **eFigure.** Effects on Withdrawal Symptoms and Changes in Carbon Monoxide Readings and Nicotine Dependence Level
eReferences

Supplement 3.
Data Sharing Statement
