# Supplementary material for: Assessment of a Text Message–Based Smoking Cessation Intervention for Adult Smokers in China: A Randomized Clinical Trial
Source: JAMA Netw Open. 2023 Mar 1;6(3):e230301. doi: 10.1001/jamanetworkopen.2023.0301 (PMC9978944; doi:10.1001/jamanetworkopen.2023.0301)
Supplement: Supplement 1. — Trial Protocol [file jamanetwopen-e230301-s001.pdf]

Design, Development and Randomized Control Trial of a Mobile Cessation intervention for Chinese adult smokers: Study Protocol

Background

Mobile health technology can promote smoking cessation and improve outcomes

Many studies have confirmed that smart phone application (app) can promote smoking cessation and improve health outcomes. Apps based on behavior-change interventions include beneficial features of short messages and internet-based interventions, but without their specific limitations. App interventions can reach out to smokers proactively, unobtrusively with personalized and confidential messages that are cost effective. Support can be offered with or without internet connection, providing effective in-the-moment help, increasing smokers involvement and tracking progress at any time. Mobile based cessation programs are increasingly part of m-health programmes promoted and supported by WHO.

Up to now, dozens of mobile tools can be used as smoking cessation support for IOS and android systems. Some limitations, however, have been identified for smokers including that: not all tools follow international smoking cessation guidelines; the content and theory used in development process is not clear; researchers cannot take advantage of previous experiences; and most studies focus on short-term impact on smoking cessation rather than longer term evidence. Currently, there is neither high quality evidence to target Chinese population nor effective mobile cessation tools that could support patients to quit from smoking.

Primary objective

We aim to develop a mobile cessation intervention based on Wechat app to support smokers to quit based on behavior-change intervention theories while taking into consideration of the context for Chinese people. After this, we will conduct a randomized controlled trial (RCT) to compare the efficacy of this tailored and behaviour change theory-based text message smoking cessation intervention with a nonpersonalized text message smoking cessation intervention.

Secondary objectives

1. To evaluate potential impact on health awareness and self-efficacy for smokers between the two kinds of interventions.

2. To identify whether certain patients are more likely to benefit from these interventions.

Methods

This study will be conducted in two steps. We firstly develop a smoking cessation intervention platform on Wechat based on TranstheoreticalModel (TTM) and Protection Motivation Theory (PMT). Both models have been independently applied to health behavior change interventions. After this, a prospective RCT using two-arm design will be conducted to assess the efficacy of this app-based treatment.

Step 1 Development of mobile cessation intervention

The design and development of this mobile cessation intervention followed three main stages.

The first stage was to develop the conceptual and theoretical framework, which was based on the interactive application of Transtheoretical Model (TTM) and Protection Motivation Theory (PMT).

TTM posits that health behavior change can be divided as six stages: pre-contemplation, contemplation, preparation, action, maintenance, and termination. It is a systematic theory of behavior change that states that health behavior is determined on a stage-to-stage basis. For any specific stage, there is intervention method to strengthen the behavior change occurrence and/or support to achieve next stage. The premise for using this theory is readiness for change. People in action, maintenance or termination stage will be normally excluded in high quality RCT design due to the homogeneity requirement.

PMT provides an important social cognitive account of protective behavior and has been extended to a more general theory of persuasive communication. There are studies applying the model to predict and evaluate protective behavior, particularly health behaviors. Applied to smoking cessation intervention, PMT theory will be used to adjust smoking behaviors' threat and coping appraisal. Behavioral intervention will be conducted from the perspective of those quitting attempt inducing factors to

enhance smokers' perception of the severity and susceptibility of smoking related diseases. The theories underlying intervention design are further described in **Table 1**.

Table 1. Conceptual and theoretical framework of Wechat application

Theory	Application	Description
TTM	Actively identify smokers' stage	Patients' willingness to quit will be assessed according to TTM. Smokers are divided as pre-contemplation, contemplation and preparation stage.
PMT	Evaluate smokers' cessation protect motivation	Develop multitask interactive function to evaluate smoking behaviors' threat appraisal and coping appraisal, including perception of the severity and susceptibility of smoking related diseases, intrinsic and extrinsic rewards, and self-efficacy, etc.
PMT	Strengthen cessation protect motivation	Personalized interventions will be provided according to demographic characteristic (such as age, gender, education), social psychological factors (such as personality, social status), structural factors (such as perception of smoking and disease) and take into consideration current smokers' cessation protect motivation level.
TTM	Provide stage-to-stage continuous support	Provide stage-to-stage support based on the systematic relationship between stages of change and processes of change. Consciousness rising, dramatic relief, environmental reevaluation strategies will be used to support smokers to reach contemplation stage;<br>Self-reevaluation strategy will be used to support smokers to reach preparation stage;<br>Self-liberation strategy will be used to support smokers to reach action stage;<br>Helping relationships, Counterconditioning, Reinforcement management, and Stimulate control strategies will be used to support smokers to reach maintenance stage.

In the second stage, we will hold several meetings. Experts in smoking cessation, health education, and IT will join the meetings and will provide comments and suggestions for the content. Then, the study team will revise those sections accordingly.

The last stage is to develop the mobile cessation tool on Wechat platform by using IT technology. We will take into account several important considerations. First, the app should ensure confidentiality of the data provided by the smokers. Second, the

system needs to be user friendly with ease for quick data entry. Third, there needs to be a back-end server that stores the data for future analysis. Fourth, it should be usable and adaptable for common operating systems. Finally, the information must be presented in a way that is easily read and interpreted. Therefore, Lime JS, an HTML5 framework for touch screens and desktop browsers, will be chosen as the basis for the development of this App. A single code base will be used for two different versions of the App: iPAD and iPhone. MySQL database will be used for keeping track of the smoker' data and behavior in the App. On the server port, with multi-tier architecture, this app is divided into display layer, business layer and data layer to improve system reliability. In addition, in view of the possible high concurrent requests, container technology is used to implement the rapid deployment strategy of multi-service instances, and a streaming media player server is established for video and audio playback to ensure fast response under the condition of high concurrent requests.

Step 2 Prospective RCT of Wechat-based intervention for smoking cessation

Enrolment procedures and eligibility criteria

Smokers will be invited to participate in this study if they fulfill the following criteria:

(1) Daily or weekly smokers

(2) aged 18 or older

(3) owned a mobile phone and used WeChat

(4) Agree to participate and sign informed consent form.

Patients will be excluded from the study if:

(1) Received any smoking cessation treatment within 30 days

(2) With HIV/AIDS or any mental diseases

(3) With other severe diseases that cannot follow doctor's advice

(4) Unwilling to participate or unable to finish the study.

Ethical consideration and informed consent

The trial was approved by the Ethics Committee of Peking University Health Science Center (IRB00001052-30063) Informed consent will be signed by all

recruited patients in their first visit.

Randomization

An independent statistician, Dr. Huaqing TAN from Guanghua School of Management, Peking University, will oversee the randomization but will not be involved in the treatment. After recruitment, participants were required to complete the baseline questionnaire and register through WeChat. With a randomized block design and the score of the Fagerström Test for Nicotine Dependence as a stratified factor, eligible participants were assigned to the intervention group or the control group. The WeChat system was also used to balance demographic characteristics. Randomization was fully computerized and automated with equal allocation. The researchers and participants were all blinded.

Baseline assessment

Research staff will conduct a face-to-face interview with recruited smokers in their first visit for baseline assessment. The interview will take approximately 15-30 minutes to complete and answers will be recorded directly into a computerized database. After completing the entire baseline assessment, participants will be given gift.

Interventions

All the participants were informed that the 8th day after randomization would be their quit day. Participants who were allocated to the intervention group received tailored and behaviour change theory-based text message smoking cessation intervention. Control group participants received a nonpersonalized text message smoking cessation intervention developed by the National Cancer Institute (NCI). It was based on well-established cognitive–behavioural cessation approaches. The details of the control group can be found elsewhere. Both groups received information by Wechat and received 1-2 messages a day for 3 months after randomization.

A Standard Protocol Item: Recommendation for Interventional Trials (SPIRIT) is presented in Table 2.

Table 2. Standard Protocol Item: Recommendation for Interventional Trials

		Follow-up (month)			
	**Baseline**	**1**	**2**	**3**	**6**
**ENROLMENT**					
Eligibility screen	X				
Informed consent	X				
Allocation	X				
**INTERVENTIONS**					
Intervention group		X	X	X	
Control group		X	X	X	
**ASSESSMENT**					
Smoking status	X	X	X	X	X
Nicotine dependence	X	X		X	X
Willingness to quit	X	X	X	X	X
Prolonged abstinence		X	X	X	X
Health awareness	X	X	X	X	X
Self-efficacy	X	X	X	X	X
Utilization of the Wechat	X	X		X	X

Follow-up visit arrangement

All participants are instructed to attend face-to-face interview with research staff 1-month, 3-month, 6-month after randomization.

At each follow-up visit, the participants will be assessed smoking status. We will also test expired air carbon monoxide at all follow-up visits.

Primary and Secondary outcome

The primary outcome was the biochemically verified 6 month sustained abstinence rate, defined as the self-report of no smoking any cigarettes after the designated quit date, validated biochemically by an expired carbon monoxide level of less than 6 ppm at each follow-up point.

Secondary outcomes are comprised of two aspects:

1.biochemically verified sustained abstinence rate at 1 month, 3 months, 1-3 months and 3-6 months, biochemically verified 24-hour point prevalence of abstinence at each follow-up point, self-reported sustained abstinence rate and 24-hour point prevalence of abstinence at each follow-up point

2.the change in nicotine dependence measured by the Fagerstrom Test for Nicotine Dependence, the change in nicotine withdrawal symptoms measured by the Minnesota Nicotine Withdrawal Scale, and the change in the readings of expired carbon monoxide.

3.Smoking cessation related health behavior: stage of change, self-efficacy, threat appraisal, coping appraisal and protection motivation.

4.Utilization of the app: time application opened, time of utilization, function used and self-report preference.

Statistical analysis

Descriptive statistics will conduct to compare the baseline status between the two groups.

Data will be analyzed on an intention-to-treat (ITT) basis. In accordance with the ITT principle, all patients will be included in the analysis, independently of the groups to which they are randomly assigned and regardless of whether they complete the treatment or not. Participants who breach the protocol (taking cessation medication or received other cessation treatment) will be followed-up in their original group and classified according to their smoking status consistent with other participants.

The Russell Standard (RS) will be used in this study. All randomized subjects will be included in the denominator for calculating abstinence rates with the exception of unavoidable loss to follow-up (died or moved to an untraceable address). Those who decline to be involved in subsequent data collection will be counted as smokers. This type of analysis is considered the most conservative and is the standard for smoking cessation studies.

The primary and secondary abstinence outcomes will analyze by logistic regression of smoking status at each timepoint. The dependent variable is smoking

status (still smoking=0, abstinent=1). The independent variable is each intervention versus the control condition. Odds ratios (Or) will used to measure the outcomes for the intervention groups compared to the control group, and 95% confidence intervals (CI) and P values will be reported to infer efficacy. In sensitivity analyses, each model will be further adjusted for baseline covariates: living area, age, education, smoking frequency and nicotine dependence at baseline.

All data will be analyzed using SPSS version 21.0. The level of bilateral significance for all statistical tests will be established at $\alpha = 0.05$.

Data management

Prof. Xiao Li from Chinese Center for Health Education will work with staff of research team to maintain quality assurance for all data. The process will be checked according to Standard Protocol Items: recommendations for interventional trials. All the data will be stored in Peking University and are password-protected. All patients' information is only accessible for personnel participating in the study. For statistical analysis, all patients' information will be encoded in order to guarantee personal information and unidentifiable during processing or reporting the result. The number of patients who drop out will be reported.

Sample size

The sample size calculation was based on the formula for a two-arm RCT. Based on earlier research, we estimated that biochemically verified continuous smoking abstinence at 6 months would be approximately 4% in the control group and 10% in the intervention groups. To achieve 80% power with a significance level of .05 (two sided), a sample size of 280 individuals was needed in each group. Assuming 20% attrition in the follow-up measurements, the total required sample size was 672.
