# Supplementary material for: Assessment of a Text Message–Based Smoking Cessation Intervention for Adult Smokers in China: A Randomized Clinical Trial
Source: JAMA Netw Open. 2023 Mar 1;6(3):e230301. doi: 10.1001/jamanetworkopen.2023.0301 (PMC9978944; doi:10.1001/jamanetworkopen.2023.0301)
Supplement: Supplement 2. — eTable. Theoretical Framework of the Intervention eMethods. Development of the Intervention Framework and the Text Bank eFigure. Effects on Withdrawal Symptoms and Changes in Carbon Monoxide Readings and Nicotine Dependence Level eReferences [file jamanetwopen-e230301-s002.pdf]

Supplementary Online Content

Lin H, Liu Y, Zhang H, Zhu Z, Zhang X, Chang C. Assessment of a text message–based smoking cessation intervention for adult smokers in China: a randomized clinical trial. *JAMA Netw Open*. 2023;6(3):e230301. doi:10.1001/jamanetworkopen.2023.0301

eTable. Theoretical Framework of the Intervention

eMethods. Development of the Intervention Framework and the Text Bank

eFigure. Effects on Withdrawal Symptoms and Changes in Carbon Monoxide Readings and Nicotine Dependence Level

eReferences

This supplementary material has been provided by the authors to give readers additional information about their work.

© 2023 Lin H et al. *JAMA Network Open*.

eTable. Theoretical Framework of the Intervention

Calendar date	Programme arm	TTM	PMT	Message bank	Example text message	Personalized information
Day 0	Registration				In the next three months, we will support you to quit smoking. Please pay attention to the information we send through WeChat. XXX will be your quit day. After that day, you should not try even a cigarette. You still have a few days to prepare.	X
Day 1-7	**Pre**quit	**W**eak quitting intention:<br>Consciousness raising<br>Dramatic relief<br>Environmental reevaluation	Increase **s**everity and **s**usceptibility	P-W and P-SS	**P-W:** Your children may imitate your smoking behaviour; quitting smoking will set a good example for your family.<br>**P-S:** Heavy work or lack of sleep lead to relapse; we encourage you to better arrange your time in the early stage of quitting.<br>**P-SS:** Half of smokers will die early from smoking.<br>**P-RR:** Quitting smoking is actually the most cost-effective health care measure.<br>**P-EE**: If there is a smoker in your home, you can encourage him or her to quit smoking together with you.	√
			Decrease **r**esponse cost, intrinsic and extrinsic **r**ewards	P-W and P-RR		√
			Increase self-**e**fficacy and response **e**fficacy	P-W and P-EE		√
		**S**trong quitting intention:<br>Stimulus control<br>Self-liberation<br>Reinforcement management	Increase **s**everity and **s**usceptibility	P-S and P-SS		√
			Decrease **r**esponse cost, intrinsic and extrinsic **r**ewards	P-S and P-RR		√
			Increase self-**e**fficacy and response **e**fficacy	P-S and P-EE		√
Day 8	Quit day				Congratulations! Today, is your quit day.<br>1. Try not to smoke after today, not even a puff.<br>2. Please remove all the tobacco products from your possession.<br>3. Avoid drinking alcohol or attending smokers party.	X
Day 9-18	Withdrawal symptom management				Please get enough sleep; you may take a nap, perform moderate exercise, take a hot bath, and drink more water.	X
Day 19-36	**E**arly quit	**R**elapse:<br>Consciousness raising<br>Dramatic relief<br>Environmental reevaluation<br>Self-reevaluation	Increase **s**everity and **s**usceptibility	E-R and E-SS	**E-R:** You can choose alternatives to help overcome the urge to smoke, such as using gum or a toothpick to keep your mouth busy.<br>**E-SS:** Compared with smoking regular cigarettes, smoking "low-tar cigarettes" does not reduce the harm of smoking.<br>**E-RR:** Currently, everyone is pursuing a healthy lifestyle; smoking is no longer a social trend.<br>**E-EE:** Your efforts are for a better life; many people like you are	√
			Decrease **r**esponse cost, intrinsic and extrinsic **r**ewards	E-R and E-RR		√
			Increase self-**e**fficacy and response **e**fficacy	E-R and E-EE		√
		Maintained abstinence:	Increase **s**everity and **s**usceptibility	E-SS		√

© 2023 Lin H et al. *JAMA Network Open*.

		No information from the TTM	Decrease **r**esponse cost, intrinsic and extrinsic **r**ewards	E-RR	quitting smoking with us now.	√
			Increase self-**e**fficacy and response **e**fficacy	E-EE		√
Day 37-90	**L**ate quit	**R**elapse:<br>Consciousness raising<br>Dramatic relief<br>Environmental reevaluation<br>Self-reevaluation	Increase **s**everity and **s**usceptibility	L-R and L-SS	**L-R:** After you maintain abstinence for a period of time, you will feel refreshed and energetic. Keep trying, you will feel it soon.<br>**L-SS:** Even occasional smoke can cause serious health problems; therefore, we need to quit smoking, not reduce the amount of smoking.<br>**L-RR**: Quitting smoking will not cause your body any problems; the possible discomfort in the early weeks of quitting is called "withdrawal symptoms," which can disappear after a few weeks.<br>**L-EE**: You may have noticed some improvements in your body, such as increased appetite and easy breathing, which are the benefits of quitting smoking.	√
			Decrease **r**esponse cost, intrinsic and extrinsic **r**ewards	L-R and L-RR		√
			Increase self-**e**fficacy and response **e**fficacy	L-R and L-EE		√
		Maintained abstinence:<br>No information from the TTM	Increase **s**everity and **s**usceptibility	L-SS		√
			Decrease **r**esponse cost, intrinsic and extrinsic **r**ewards	L-RR		√
			Increase self-**e**fficacy and response **e**fficacy	L-EE		√

© 2023 Lin H et al. *JAMA Network Open*.

eMethods. Development of the Intervention Framework and the Text Bank

Development of the intervention framework

The first stage was to develop the theoretical framework of the intervention based on the transtheoretical model (TTM) and protection motivation theory (PMT). Both models have been independently applied to health behaviour change interventions [1-2]. For the TTM, given the systematic relationship between the stages and processes of change, several strategies were used to strengthen behaviour change and/or to achieve the next stage. On the prequit date, messages related to consciousness raising, dramatic relief, and environmental reevaluation were provided to smokers with weak quitting intention. Messages related to stimulus control, self-liberation, and reinforcement management were provided to smokers with strong quitting intention. On the early quit and late quit dates, messages related to consciousness raising, dramatic relief, environmental reevaluation and self-reevaluation were sent to smokers who had relapsed. Smokers who remained abstinent only received information based on PMT. To evaluate smokers' quitting intention, we used a 5-point scale ranging from not at all likely (1) to very likely (5) regarding the likelihood that they would try to quit in the next 6 months [3]. (Not all the participants had strong quitting intention. Some smokers did not want to quit smoking and only intended to reduce the amount of smoking or were curious about what a mobile cessation intervention is. Therefore, they expressed a desire to join this study, but may not have been at all likely to quit in the next six months)

The WeChat application evaluated the PMT construct score by asking questions and recording information; then, it automatically calculated the lower score of the subcontent that needed to be strengthened. Specifically, the scale comprised 21 items using a 7-point Likert-type scale with responses ranging from 1 (definitely disagree) to 7 (definitely agree). Each construct subscale includes three items, and we computed the mean as the subscale score. We have published the details of this scale and evaluation process elsewhere [4-5]. A more detailed framework of the text and intervention based on the TTM and PMT is shown in Supplemental Table 1.

Development of the text bank

The second stage was to develop the intervention message bank. Messages were developed by Peking University, School of Public Health, with the input of smokers and smoking cessation professionals. Most of the core information came from official reports and publications, such as China reporting the health hazards of smoking, clinical guidelines for smoking cessation intervention in China, WHO reports on treating tobacco dependence in primary care and published research articles. An expert committee consisting of health education experts, smoking cessation experts, social medicine experts and IT experts was established. The committee assessed the quality of the messages, including whether the messages were scientific, motivating and acceptable.

The messages had a three-layer framework. The first layer was divided based on time and consisted of the prequit message (1-7 days), quit day message (8 days), withdrawal symptom management message (9-18), early quit message (19-36 days), and late period message (37-90

© 2023 Lin H et al. *JAMA Network Open*.

days). The second layer was divided based on the TTM. Before the quit day, messages were classified as 1. strong quitting intention or 2. weak quitting intention. After the quit day, messages were classified as 1. maintained abstinence and 2. relapsed. The third layer was divided based on PMT. Messages were classified as 1. increased severity and susceptibility; 2. decreased response cost and intrinsic and extrinsic rewards; and 3. increased self-efficacy and response efficacy. The core motivational messages consisted of 14 subgroups with a total of 200 text messages. There were also approximately 200 contact messages.

The last stage was to deploy the message library on the WeChat platform by using information technology. Our IT team completed the development process with several important considerations. First, the application needed to ensure confidentiality of the data. Second, the system needed to be user-friendly with ease for quick data entry. Third, there needed to be a back-end server to store the data. Fourth, the information needed to be presented in a way that could be easily read and interpreted.

© 2023 Lin H et al. *JAMA Network Open*.

eFigure. Effects on Withdrawal Symptoms and Changes in Carbon Monoxide Readings and Nicotine Dependence Level

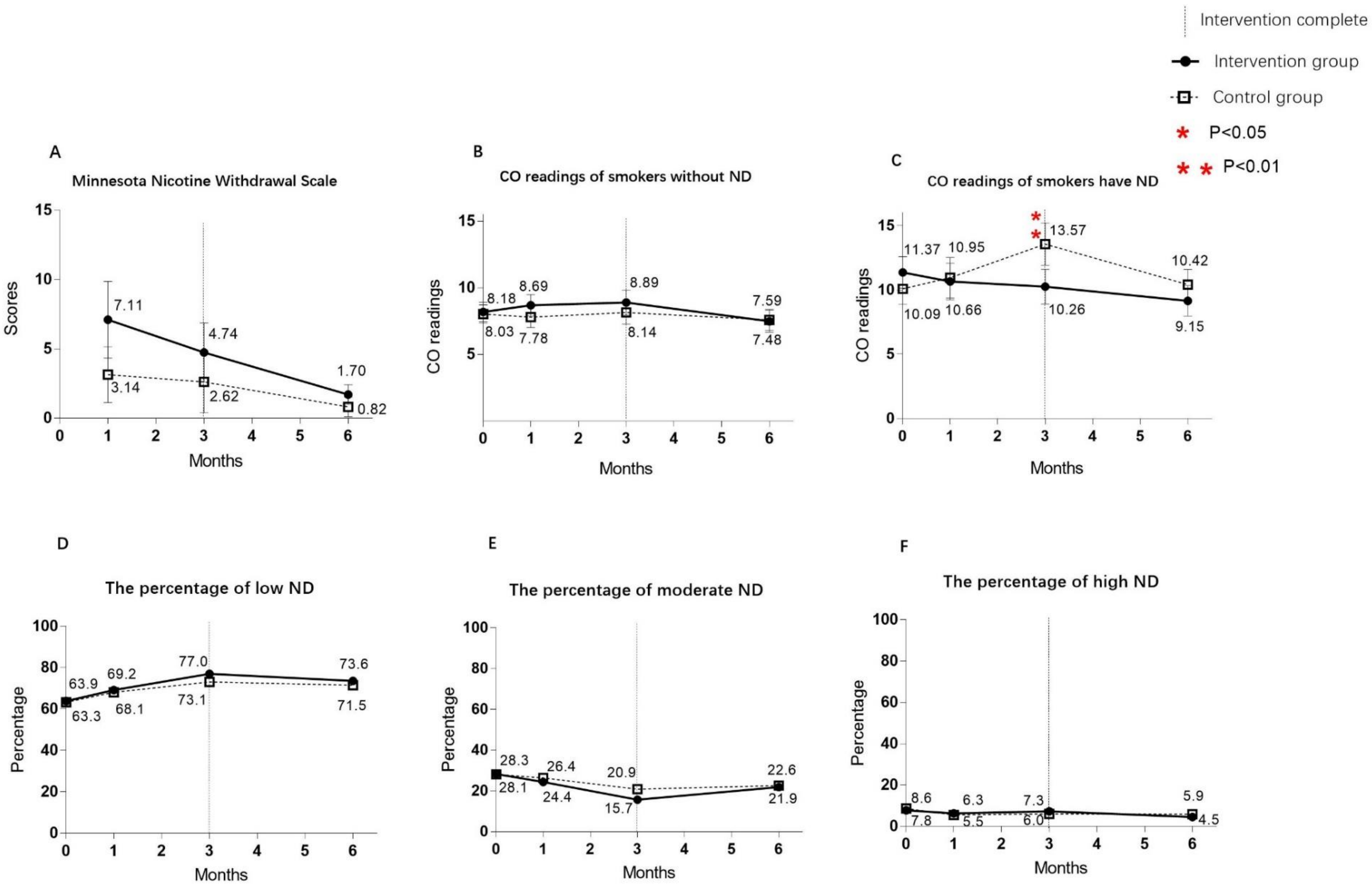

The score of withdrawal symptoms deceased from months 1 to 6, and we did not detect any significant differences between the two groups. When the sample was stratified by ND, CO readings were similar between the two groups for smokers without ND. Interestingly, we found that the intervention group reported significantly lower CO readings at 3 months for nicotine-dependent smokers. Regarding the change in ND, we found that the proportion of each ND group (low/moderate/high) remained steady throughout the whole period and was not significantly different between the two groups (Supplemental Figure 1).

© 2023 Lin H et al. *JAMA Network Open*.

eReferences

1. Chawla G, Kansal AP, Deokar K, et al. Effects of Stage-matched Repeated Individual Behavioural Counselling Session (RIBCS) as an Intervention for Decreased and Stopping Smoking. Monaldi Archives for Chest Disease, 2020, 90(1):119-125.

2. Sabzmakan L, Ghasemi M, Jafarabadi MA, et al. Factors Associated with Tobacco Use Among Iranian Adolescents: An application of Protection Motivation Theory. Substance Use & Misuse, 2018, 53(3):1-8

3. Biener L, Abrams DB. The Contemplation Ladder: validation of a measure of readiness to consider smoking cessation. Health Psychol. 1991;10(5):360-365.

4. Huang CL, Lin HH, Wang HH. The psychometric properties of the Chinese version of the Fagerstrom Test for Nicotine Dependence. Addict Behav. 2006;31(12):2324-2327.

5. Lin HX, Chang C. Factors associated with the quitting intention among Chinese adults: Application of protection motivation theory. Current Psychology, 2021 Feb 23.

© 2023 Lin H et al. *JAMA Network Open*.
