# Supplementary material for: Assessment of a Text Message–Based Smoking Cessation Intervention for Adult Smokers in China: A Randomized Clinical Trial
Source: JAMA Netw Open. 2023 Mar 1;6(3):e230301. doi: 10.1001/jamanetworkopen.2023.0301 (PMC9978944; doi:10.1001/jamanetworkopen.2023.0301)
Supplement: Supplement 3. — Data Sharing Statement [file jamanetwopen-e230301-s003.pdf]

Data Sharing Statement

Lin. Assessment of a Text Message-Based Smoking Cessation Intervention for Adult Smokers in China. *JAMA Netw Open*. Published March 01, 2023.
doi:10.1001/jamanetworkopen.2023.0301

Data
Data available: No
